# Effects of Copper Oxide Nanoparticles on Paddy Soil Properties and Components

**DOI:** 10.3390/nano8100839

**Published:** 2018-10-16

**Authors:** Jiyan Shi, Jien Ye, Huaxiang Fang, Shu Zhang, Chen Xu

**Affiliations:** 1 Department of Environmental Engineering, College of Environmental and Resource Sciences, Zhejiang University, Hangzhou 310058, China; shijiyan@zju.edu.cn (J.S.); yejien@zju.edu.cn (J.Y.); 21414041@zju.edu.cn (H.F.); 21414071@zju.edu.cn (S.Z.); 2 MOE Key Laboratory of Environment Remediation and Ecological Health, College of Environmental & Resource Science, Zhejiang University, Hangzhou 310058, China; 3 Zhejiang Bestwa EnviTech Company Limited, Hangzhou 310015, China

**Keywords:** CuO nanoparticles, paddy soil, soil physicochemical properties, Cu form transformation

## Abstract

The wide use of metal-based nanoparticles (MNPs) will inevitably lead to their release into soil, and consequently affect the quality and ecological functions of soil environments. In this study, two paddy soils with different properties were exposed to CuO NPs to evaluate the transformation of CuO NPs and their effects on soil properties and components. The results of single chemical extraction and X-ray absorption fine structure analysis showed that CuO NPs could release Cu ions once being applied into the flooding paddy soil and then progress toward the more stable forms (Cu_2_S and Cu(OH)_2_). CuO NPs could change the soil properties by increasing the pH and Eh of the lower organic matter-soil rather than those of the higher organic matter-soil. Furthermore, we found that the 1000 mg/kg CuO NPs could accelerate the degradation or mineralization of the organic matter, as well as the Fe reduction process, by increasing the Fe(II) content by 293% after flooding for 60 days in the lower organic matter soil. The microbial biomass in both soils was severely inhibited by CuO NPs and the organic matter could partly mitigate the negative effects of CuO NPs.

## 1. Introduction

With the rapid development of nanotechnology, metal-based nanoparticles (MNPs), which have unique electrical, magnetic and catalytic properties compared with conventional materials, are increasingly being used in industrial production and daily necessities [1]. As one of the most widely used MNPs, CuO nanoparticles (CuO NPs) have been widely used in semiconductor devices, industrial catalysts, and antimicrobial preparations [2]. During the process of nanomaterial production, transportation, usage and disposal, MNPs are inevitably released to air, water and soil. Although it is still difficult to accurately measure MNPs concentrations in soil, the exposure calculator model suggests that soil could be the major sink of MNPs rather than water and air [3,4,5].

Soil plays an important role in substance and energy exchange in the ecosystem, and its quality and safety are increasingly being considered as essential for the balance and stability of the entire ecosystem. The maintenance of soil function depends on the physical, chemical and biological properties of the soil, including texture, structure, chemical composition, temperature, humidity, pH, redox potential and organic content [6]. These different characteristics affect soil fertility, organic synthesis and degradation, nutrient supply, trace elements bioavailability, and soil biological metabolism. Notably, soil contaminations (As, Ba, Cd, Cu, Pb, Sr) were found to be strongly linked to the soil redox potential (Eh) and the chemistry of dissolved organic carbon (DOC), Fe, and Mn in different rice paddy soils originating from the United States, Europe and Asia [7,8,9]. As a new type of non-degradable contaminant, MNPs have caused concerns due to their potential risks to the soil environment [10,11]. On the one hand, the migration and transformation of MNPs can be affected by the chemical property of the soil liquid phase and surface features of the soil solid phase. The aggregation of TiO_2_ NPs was found to be positively correlated with soil dissolved organic carbon and clay content but was negatively correlated with ionic strength, pH and zeta potential [12]. Humic acid was also found to stabilize nanoparticle suspensions [13]. On the other hand, MNPs can also affect the components and physicochemical properties of soil [11]. CuO NPs were reported to significantly decrease soil redox potential but increase electrical conductivity [14]. Slight impacts on soil microbial biomass and community structures were also observed when paddy soil was combined with TiO_2_ NPs [15]. However, studies on the long-term interactions between MNPs and soil properties are still lacking.

Paddy soil is the most widespread and typical agricultural soil in China, the quality and safety of which directly affects the grain yield and the national economy. Due to the usage of sewage sludge, nano-related pesticides, and fertilizers in agriculture, paddy soil is more likely to be exposed to MNPs [16]. Moreover, paddy soil has a periodically flooding–drying water management, leading to a constantly changing redox potential in the soil environment. Researches showed that the physicochemical properties of paddy soil are more sensitive to the external environment than other farmlands [17], and our previous research also suggested that the response of the microbial community in paddy soil is different from that in dryland soil [15]. However, the knowledge of how CuO NPs affect paddy soil properties and components is still limited.

In this study, two paddy soils with different properties were exposed to different dosages of CuO NPs, and a complete flooding–drying cycle during rice cultivation was simulated. The main aims were to (1) explore the transformation of CuO NPs in paddy soil during a flooding–drying cycle; and (2) investigate the effects of CuO NPs on the properties and components of different paddy soils. This study endeavors to contribute to the assessment of environmental and ecological risks of MNPs to the paddy soil environment.

## 2. Materials and Methods

### 2.1. Soils

Two typical paddy soils were sampled from sites in Jingshan (JSS) town, Hangzhou, South China (119°51′ E, 30°22′ N) and Heihe (HHS), Heilongjiang, North China (127°46′ E, 50°24′ N). The upper layer (0–20 cm) of the soil was collected, and after removal of visible stones, branches and roots, soil samples were air-dried and sieved to less than 2 mm. The contents of organic matter in JSS and HHS were 4.15% and 8.04%, respectively (Supporting Information (SI) Table S1).

### 2.2. CuO Particles

Both CuO NPs and CuO bulk particles (BPs) properties were previously measured and reported [18]. CuO NPs (Nachen Sci. &Tech. Ltd., Beijing, China) have a specific surface of 131.0 cm^2^/g with a purity of >99.9%. The hydrodynamic diameter of CuO NPs in Mill-Q water is 240.0 nm. CuO bulk particles (Sinopharm Chemical Reagent Co., Ltd., Shanghai, China) have an average particles size of 1346 nm. The hydrodynamic diameter of CuO BPs in Mill-Q water is over 1 μm.

### 2.3. Soil Culture Experiment

In this study, the target concentrations were 10, 100, and 1000 mg/kg for CuO NPs and 1000 mg/kg for CuO BPs as a comparison with CuO NPs. The unspiked air-dried soil was set as a control. Though the average concentration of CuO NPs in the real environment is expected to be at lower levels, a high concentration of CuO NPs may exist in some special regions. All culture experiments were conducted in glass bottles (height 190 mm, diameter 90 mm) in phytotron. Each bottle contained 0.5 kg of soil and 400 mL of deionized water to achieve water-saturated soil and maintain the depth of water layer over the soil surface for 5 cm. During the cultivation, deionized water was added every two days to keep the water level consistent. After flooding treatment for 60 days, the soils entered into the intermittent flooding–drying process until drying in 90 days. The soils were sampled on 0 (10 h), 10, 30, 60 and 90 days. After the culture experiment, the rest of the soil was freeze-dried for 72 h and stored in a vacuum drier. Every group had three replicates.

### 2.4. Analysis of Soil Properties and Components

The properties and components of the soil determined included pH, Eh, total organic carbon (TOC), DOC, ferrous ion (Fe^2+^), and microbial biomass carbon (MBC).

Soil pH and Eh. The initial soil pH was measured by a pH meter with a soil/solution ratio of 1:2.5. During the soil culture experiment, the soil pH and Eh were measured by an ion analyzer (Thermo-Orion, Beverly, MA, USA) with a pH electrode and an oxidation reduction potential electrode.

Total organic carbon (TOC). A certain weight of moist soil was dried at 105 °C for 24 h and the dried soil was put into a muffle furnace at 400 °C for 8 h to calculate the TOC.

Dissolved organic carbon (DOC). Moist soil (2.5 g) was extracted by 25 mL 0.5 mol/L K_2_SO_4_ for 30 min. After being centrifuged at 6000× *g* for 10 min, the supernatant was determined by the Total Organic Carbon Analyzer TOC-V/CPN (Multi N/C 2100, Analytik Jena, Jena, Germany).

Ferrous ion (Fe^2+^). Moist soil (0.5 g) was extracted by 5 mL 0.5 mol/L HCl for 24 h and centrifuged at 2000× *g* for 5 min. One milliliter of filtrate was mixed with 5 mL of sodium acetate buffer and 5 mL 0.1% phenanthroline and the mixture was measured using a spectrophotometer at λ = 510 nm after a 30 min color reaction.

Microbial biomass carbon (MBC). The fumigation-extraction method was used to determine the MBC [18,19,20]. Ten grams of moist soil were divided into two equal portions and one portion was incubated with ethanol free CHCl_3_ in the dark for 24 h. After incubation, the soils were extracted with 25 mL 0.5 mol/L K_2_SO_4_ for 30 min and the extraction was reserved after centrifugation and filtration. The fumigated portion was removed of CHCl_3_ by boiling water bath for 1 h. The total organic carbon concentration of the extraction was determined by Total Organic Carbon Analyzer TOC-V/CPN (Multi N/C 2100, Analytik Jena, Jena, Germany). The MBC was calculated by the following equation [21]:

MBC = (C_f_ − C_nf_)/k_EC_, (1)

where C_f_ is the organic carbon extracted from fumigated soil, C_nf_ is the organic carbon extracted from non-fumigated soil, and k_EC_ is 0.45.

### 2.5. Analysis of Cu Bioavailability

The bioavailability of Cu was determined by single chemical extraction and NH_4_NO_3_ extraction [22,23,24,25]. The freeze-dried soil was extracted by 0.01 mol/L CaCl_2_ (1:5 *w*/*v*, 2 h), and 0.05 mol/L EDTA (1:5 *w*/*v*, 1 h), respectively. Microbial available Cu (Cu-mic) was measured with a CHCl_3_ fumigation procedure similar to the MBC measurement, except for the extractant changing into NH_4_NO_3_ [25]. The Cu concentration of the extractions was determined by an atomic absorption spectrometer (AAS, MKII M6, Thermo Electron, Waltham, MA, USA). The Cu-mic was calculated by the following equation:

Cu_mic_ = Cu_f_ − Cu_nf_, (2)

where Cu_f_ is the Cu concentration in the fumigated extraction, and Cu_nf_ is the Cu concentration in the nonfumigated extraction.

### 2.6. Synchrotron Radiation X-ray Absorption Fine Structure (XAFS) Analysis

Synchrotron radiation XAFS was used to characterize the chemical forms of Cu in soil. The samples were prepared and operated according to Cheng’s method [14]. Briefly, the lyophilized soils were pressed into slices and placed on sample holders by tape. The Cu K-edge XAFS spectra of the samples and references were recorded in beamline 14W1 at the Shanghai Synchrotron Radiation Facility (SSRF, Shanghai, China). The spectra were processed by IFEFFIT Athena software to determine the main forms of Cu in soil. Details on the K-edge XAFS and data analysis are presented in the SI.

### 2.7. Statistical Analysis

SPSS version 19.0 software was used to perform one-way analysis of variance (ANOVA). The significance levels (*p* < 0.05) between the different treatments and control were determined by the Fisher’s least significant difference (LSD) test.

## 3. Results

### 3.1. Transformation of CuO NPs in the Paddy Soil

**Figure 1:**
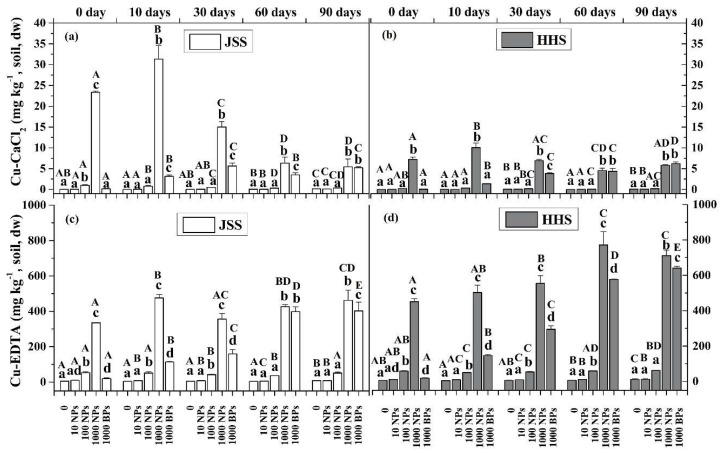
Cu content in the soil extracted by CaCl_2_ (**a**,**b**), and EDTA (**c**,**d**). White: Jinshan Soil (JSS); Gray: Heihe Soil (HHS). Error bars indicate the standard deviation of the mean (*n* = 3). Lowercase letters indicate the significance between the different dose exposure treatments over the same period. Uppercase letters indicate the significance between the different time treatments of the same exposed dosage (*p* < 0.05).

**Figure 2:**
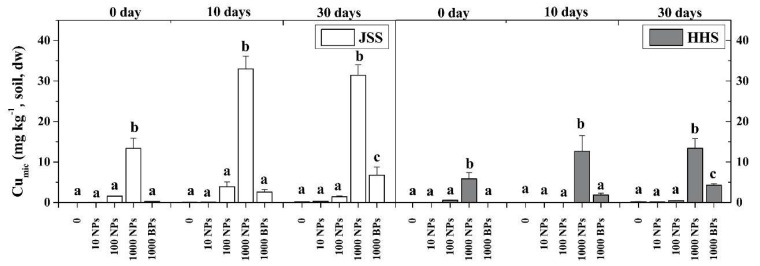
The available microbial Cu in two tested soils during flooding period. White: Jinshan Soil (JSS); Gray: Heihe Soil (HHS). Error bars indicate the standard deviation of the mean (*n* = 3). Lowercase letters indicate the significance between the different dose exposure treatments over the same period (*p* < 0.05).

The different bio-availabilities of CuO NPs in the two tested paddy soils are shown in Figure 1 and Figure 2. The CaCl_2_-extractable Cu (Cu-CaCl_2_) is regarded as the water-soluble form, the EDTA-extractable Cu (Cu-EDTA) is the exchangeable form, and the NH_4_NO_3_ extractant is considered to be the microbial available Cu (Cu-mic). The contents of Cu-CaCl_2_ and Cu-EDTA were significantly in both soils increased by the addition of higher concentrations of CuO NPs (100 and 1000 mg/kg) (Figure 1). Obviously, the CuO NPs immediately released ions and reacted with soil substances within 10 h. The content of Cu-CaCl_2_ in JSS was always higher than that in HHS during the whole culture experiment, which peaked in 10 days, being 890 times higher than the control for 1000 mg/kg CuO NPs treatment in JSS and 559 times higher in HHS. With the flooding time prolonged further, the content of Cu-CaCl_2_ in highly exposed soil decreased gradually to only 101 times and 31 times more than the control in JSS and HHS, respectively (Figure 1a,b). The content of Cu-EDTA kept increasing gradually as flooding time extended (Figure 1c,d). The Cu-EDTA in the soils exposed to 1000 mg/kg CuO NPs reached a peak of 425.7 mg/kg (JSS) and 771.2 mg/kg (HHS) after flooding for 60 days. The alternation of drying and wetting showed slight effects on the content of both Cu phases. The microbial available Cu (Cu-mic) in two tested soils was determined in the first 30 days (Figure 2). The content of Cu-mic in HHS was higher than that in JSS, especially in the treatment of 1000 mg/kg CuO NPs. All the three forms of Cu content in the CuO BPs group increased slightly when flooding time was prolonged and held steadily after drying. Despite the significant lower bioavailability of CuO BPs than CuO NPs in the early period (0–30 days), a similar changing trend in CuO NPs groups was also observed in the treatment of CuO BPs (Figure 1 and Figure 2).

**Figure 3:**
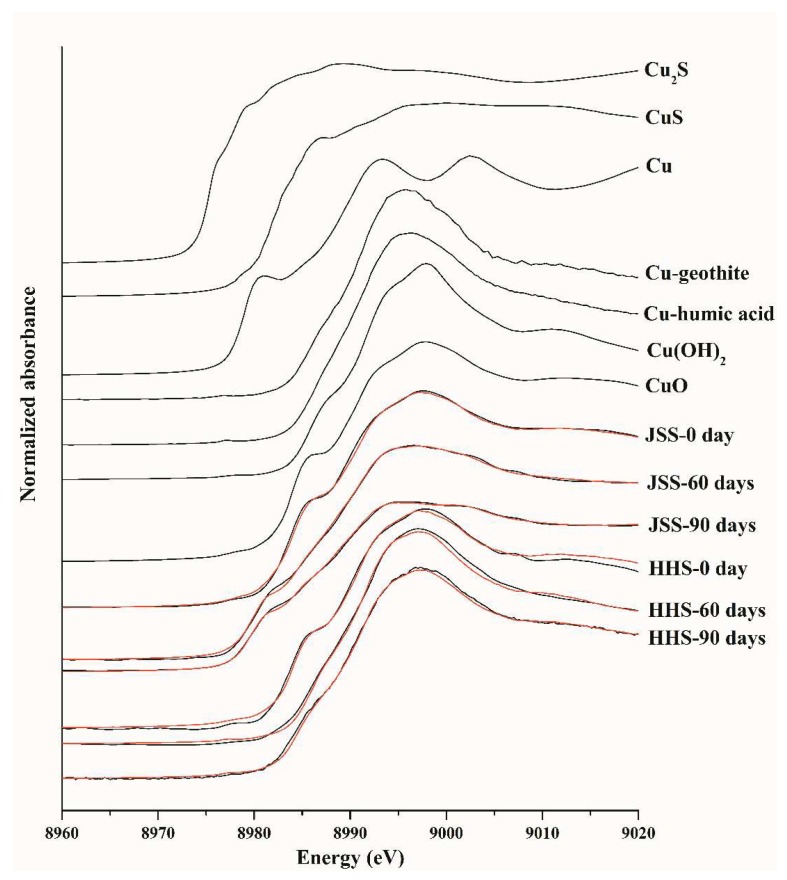
Comparison of Cu K-edge XANES spectra of reference compounds and different soil samples exposed to 1000 mg/kg CuO NPs. Red lines are the linear fitting results.

Synchrotron radiation X-ray absorption fine structure (XAFS) was used to characterize the molecular speciation changes of Cu in the 1000 mg/kg CuO NPs treated soils. Linear combination fitting results showed that CuO, Cu combined with humic acid (Cu-humic acid), Cu_2_S and Cu were the main forms of Cu in the tested soils (Figure 3). The fitting results are shown in Table 1. Once applied to the soil, the CuO NPs mainly remained in the form of CuO, with portions combined with goethite and sulfur element in the soils and transformed into Cu-goethite and CuS. After flooding for 60 days, most of the Cu element in JSS was reduced to Cu_2_S (38.7%) and Cu (27.5%), and the rest was Cu adsorbed on humic acid (24.9%) and Cu(OH)_2_ (11.1%), while the majority of Cu in HHS transformed to Cu adsorbed on humic acid (64.1%) and Cu(OH)_2_ (39.9%). After 90 days in the drying condition, the Cu element in JSS was further reduced, while the major forms in HHS were still Cu-humic acid (43.7%) and Cu(OH)_2_ (19.0%), with 41.9% transformed to CuO.

**Figure 4:**
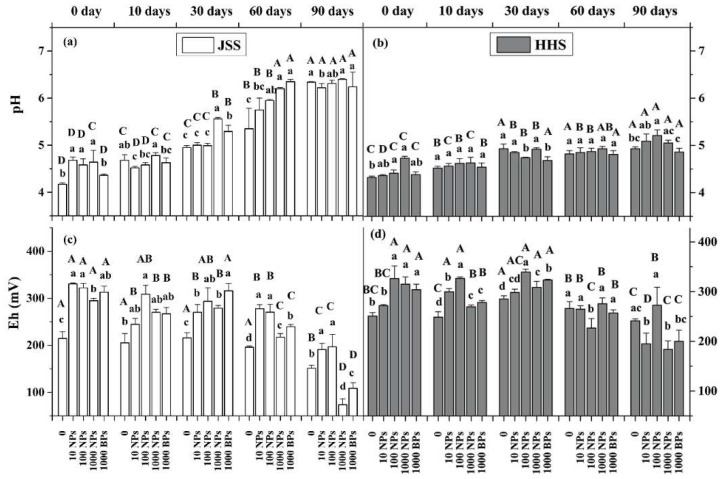
pH and Eh of two tested soils during flooding–drying period. White: Jinshan Soil (JSS); Gray: Heihe Soil (HHS). (**a**,**b**) pH; (**c**,**d**): Eh (mV). Error bars indicate the standard deviation of the mean (*n* = 3). Lowercase letters indicate the significance between the different dose exposure treatments over the same period. Uppercase letters indicate the significance between the different time treatments of the same exposed dosage (*p* < 0.05).

CuO NPs had an acute effect on soil pH within 10 h of exposure (Figure 4a,b). All dosages of CuO NPs significantly increased the soil pH of JSS, and the pH of HHS increased when the soil was exposed to higher concentrations of CuO NPs (>100 mg/kg). CuO BPs showed no significant effect on the pH in the first 10 days. With the flooding time extended, the pH of JSS went up to 5.3 on 60 days, showing a significant dose-response relationship, while the CuO NPs showed no significant effect on the pH of HHS. After two tested soils were dried (90 days), the pH of JSS greatly increased to 6.3 with no significant difference among treatments, while the pH of HHS remained steady during the flooding periods. Although the effect of CuO BPs on pH in JSS lagged behind CuO NPs, there was still a time-effect relationship, and the gap between CuO BPs and CuO NPs disappeared after flooding for 60 days. However, CuO BPs had no significant effect on the pH of HHS, even when the soil had been flooded for 60 days.

The Eh values of the two tested soils in the control groups showed no significant change during the flooding time, while the amendment of CuO NPs induced an acute and evident increase in Eh values (Figure 4c,d). Notably, CuO BPs also increased Eh in the initial periods. After 60 days of flooding, Eh in all treatments decreased obviously, and 1000 mg/kg CuO NPs showed significantly lower effect on the enhancement of Eh in JSS. Moreover, after undergoing the alternation of drying and wetting (90 days), the Eh had dramatically decreased with the treatment of 1000 mg/kg CuO NPs, especially in JSS groups.

### 3.2. Effect of CuO NPs on Soil Organic Matter

**Figure 5:**
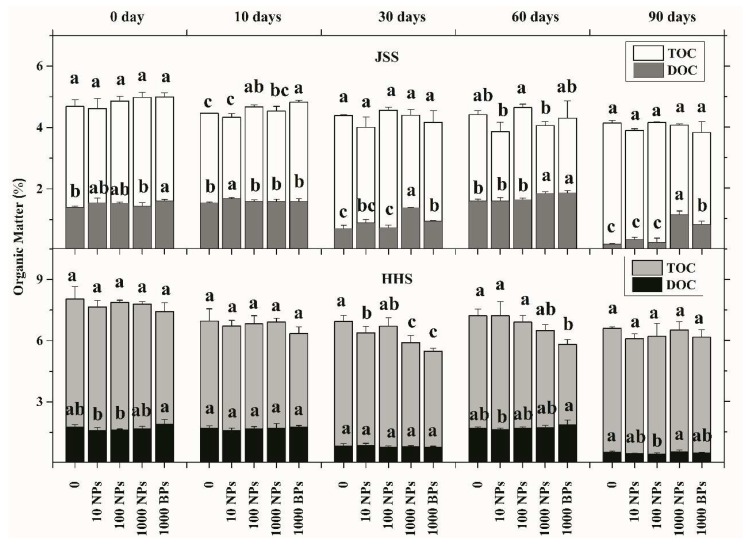
The total organic matter (TOC) and dissolved organic matter (DOC) of two tested soils during flooding–drying period. White: Jinshan Soil (JSS) TOC; Light Gray: Heihe Soil (HHS) TOC; Dark Gray: Jinshan Soil (JSS) DOC; Black: Heihe Soil (HHS) DOC. Error bars indicate the standard deviation of the mean (*n* = 3). Lowercase letters indicate the significance between the different dose exposure treatments over the same period (*p* < 0.05).

The TOC and DOC of the two tested soils during the flooding–drying period were determined and the results are shown in Figure 5. The TOC of JSS had little fluctuation during the whole cultivation period, while the content of DOC exposed to 1000 mg/kg CuO NPs was about two times higher than the control after flooding for 30 days. Similarly, the amendment of CuO BPs increased DOC content by 32.5%. The alternate drying-wetting further widened the gap between the 1000 mg/kg treatments and the control. The carbon changes in HHS showed different trends compared to JSS. In the early stages (0–10 days), CuO NPs and CuO BPs showed no effects on either the TOC or the DOC of HHS. With the extended flooding time, the content of TOC decreased with the increasing CuO NPs, as well as in the CuO BPs treatment. However, the impacts of CuO NPs and CuO BPs on TOC faded after the process of wetting and drying. The amendment of CuO NPs and CuO BPs had no significant effect on the content of DOC in HHS.

### 3.3. Effect of CuO NPs on Soil Fe(II) Content

**Figure 6:**
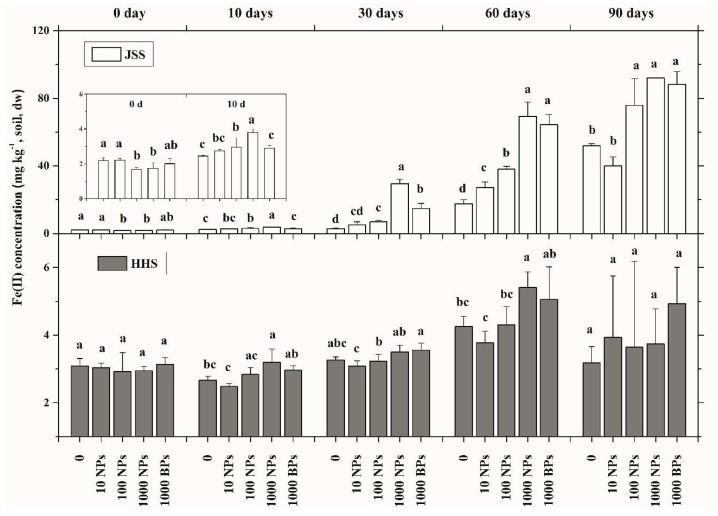
The Fe(II) of two tested soils during flooding–drying period. White: Jinshan Soil (JSS); Gray: Heihe Soil (HHS). Error bars indicate the standard deviation of the mean (*n* = 3). Lowercase letters indicate the significance between the different dose exposure treatments over the same period.

The contents of Fe(II) in the two tested soils were determined during the flooding–drying period (Figure 6). The initial concentrations of Fe(II) in JSS and HHS were 1.97 and 3.03 mg/kg, respectively (average data on 0 day). With the extension of flooding, Fe(II) in JSS increased rapidly in a dose and incubation time-dependent manner. Fe(II) in JSS after 60 days of flooding increased to 43.35 (data range from 14.82 to 62.65) mg/kg, which continually raised to 67.45 (data range from 53.04 to 92.27) mg/kg after the alternation of drying and wetting. Fe(II) in HHS showed a slight increase during the flooding period, while the alternate drying–wetting induced a significant decrease of Fe(II) content. Moreover, the effect of CuO BPs on the Fe(II) content was similar but lower than the same dosage of CuO NPs.

### 3.4. Effect of CuO NPs on Soil Microbial Biomass Carbon

**Figure 7:**
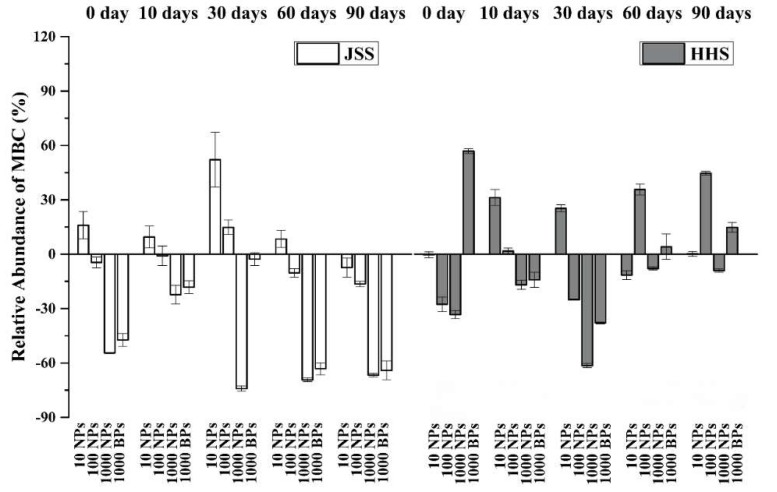
The relative abundance of microbial biomass carbon (MBC) in two tested soils (compared to the control). White: Jinshan Soil (JSS); Gray: Heihe Soil (HHS). Error bars indicate the standard deviation of the mean (*n* = 3).

The changes of microbial biomass in soil could be represented by microbial biomass carbon (MBC) (Figure 7). In general, low doses of CuO NPs increased the content of MBC to some degree, while high doses of CuO NPs resulted in a severe decrease of MBC. In JSS groups, the amendment of 1000 mg/kg CuO NPs decreased the amount of the MBC significantly, and the inhibitory effect of a high concentration CuO NPs got stronger as the treatment time extended. The negative effect of 1000 mg/kg CuO NPs on MBC in HHS kept rising in the first 30 days, but was obviously mitigated in the late stages. Besides, CuO BPs also induced a decrease in MBC of both soils with lower effects than the same dosage of CuO NPs.

## 4. Discussion

Once introduced into soil, MNPs break down thermochemically with the ions released from the inner core of the particle into the soil solutions, and the dissolution of MNPs occurs rapidly, especially in acidic conditions [26,27]. In this study, copper ions were dissolved out from CuO NPs in a short time (10 h) and the difference of CuO NPs’ solubility in the two soils depended on the soil properties. Soil organic matters were found to be one of the most important factors affecting the environmental behaviors of metal-based nanoparticles [28]. In our study, the HHS had more than twice the organic matter content than the JSS, and consistently, the much higher extractable Cu and Cu-humic acid were detected in HHS, while the content of soluble Cu in soil was oppositely correlated with the soil organic matter content. Previous studies have proposed that the combined between humic acid and ZnS NPs increased the stability of nanoparticles in suspension liquid by electrostatic repulsion and steric hindrance [29]. Additionally, the adhesion of organic matter to nanoparticles prevents further dissolution of metal ions [30,31]. In addition to this, the metals already present in soils were reported to affect the adsorption behavior and availability of nanomaterials, and the interactions between nanoparticles and pre-existing contaminants can affect their toxicity, bioaccumulation and risk in the environment [32,33]. Most metal ions dissolved out from MNPs tend to combine with the abundant charged mineral substances, organic matter and microorganisms in soil [34], which might explain the result of CuO NPs transforming into more stable precipitations and complexes as the time extended. Compared to the soluble Cu, the organic associated forms of Cu showed a positive correlation with the soil organic matter content, which might be due to the abundant functional groups and strong complexing capacities of organic matter [35]. Cu-mic was determined to represent the available Cu that can be taken in by microorganisms. The similar changing of Cu-mic with that of soluble Cu indicated that the Cu ions dissolved out from CuO NPs might be taken in by microorganisms and consequently resulting in toxicity. Furthermore, the trade-off between the soluble Cu and exchangeable Cu in the two soils could explain the difference in the microbial biomass of different soils. Moreover, the CuO BPs showed lower bioavailability and weaker and hysteretic effects on microbial toxicity, due to their larger size but smaller specific surface area compared to CuO NPs [36].

Proton activity (pH) in soil is one of the most essential physicochemical properties and is closely related to the availability of soil nutrients, microbial activity and plant growth and development. Cullen found that the amendment of nanoscale zero valent iron (NZVI) in soil could significantly increase the pH value of a soil solution [37], which was consistent with our results of soil pH increasing under CuO NPs treatment. CuO NPs can consume H^+^ in soil solution, especially in acidic soils, to produce Cu ions and Cu(OH)^+^, which consequently increases the soil pH. Reports have demonstrated that metal ions can dissolve out from MNPs in acidic soil in a short time [27], which was confirmed by our study of the Cu transformation, therefore leading to the acutely and dramatically increasing response of pH values in both tested soils once exposed to CuO NPs. The diameter of the CuO BPs was 30 times less than that of the CuO BPs, meaning that there was much lower reactivity of CuO BPs which consequently led to a lower and hysteretic effect on the soil pH than CuO NPs. Moreover, the soil organic matter could interfere with the interactions between nanoparticles and soil substances through adsorption or coating to the surface of MNPs and limiting the mobility of nanoparticles [38], resulting in the less significant increasing of pH in HHS with high organic matter content than that in JSS with low organic matter content.

Redox potential indicating the oxidation-reduction state of soil is meaningful to paddy soil. High soil aeration induces excessive consumption of soil organic matter, while poor soil aeration accelerates the accumulation of Fe(II), Mn(II), and H_2_S and interferes with the root respiration of rice, and promote the production of greenhouse gases as well. In the flooded paddy soil, the air oxygen diffusion is blocked by the water layer; meanwhile, the soil microorganisms continuously consume oxygen in soil and produce reductive substances, resulting in a gradual decrease in the soil redox potential [39]. However, due to the mini-system in this study that had less soil quantity and flooding depth compared to the actual environment, the Eh in the two tested soils was quiet slowly reduced. Frenk found that 1% CuO NPs would reduce the soil Eh, while after sterilization, the amendment of CuO NPs raised the Eh by the consumption of H^+^ [40], which means that the CuO NPs have a double effect on the Eh through the positive chemical mechanism and the negative microbial mechanism. In this study, we found that the exposure of CuO NPs could increase the Eh significantly in both soils, which might result from the lower microbial biomass and activity in the flooding soil system than the dryland soil and the rhizosphere soil system [14,41], and also the strong inhibitory effect of CuO NPs on soil enzyme activities and microbial diversity in paddy soil [15]. The rebounding Eh in the drying condition treated with CuO NPs, as well as the similar reaction of Cu BPs with CuO NPs, have verified the above viewpoints.

Iron is an important variable-valence element in the redox system. In anaerobic environments, Fe(III) can be chemically reduced by humus, hydroquinone and some low-molecular-weight organic acids. However, the majority of Fe(III) is considered to be reduced by the microbial dissimilatory Fe(III) reduction process, in which an electron from the organic matter as the electron donor is transferred to Fe(III) as an electron acceptor via microbial extracellular respiration [42,43]. The microbial dissimilatory Fe(III) reduction is vital to the flooding paddy soil ecosystem as it affects the cycle of carbon–nitrogen–sulfur, the degradation of pollutants and the production of methane [44,45]. With the prolonged flooding time, the Eh of soil reduces accordingly, resulting in the increase of reducing substances and the activity of iron-reducing bacteria in soil. Thus, the Fe(III) in the two tested soils could be simultaneously reduced by both chemical and microbial ways. Many studies have shown the complex interactions between Cu(II) and Fe(II) in different systems [46,47,48]. In the flooding paddy soil, Fe(II) from dissimilatory iron reduction could react with Cu(II) to form ferrihydrite and Cu_2_O, respectively, and Cu(I), as a reductant, could further reduce the Fe(III) and Fe(II) [49]. Our research has demonstrated the improvement of Fe(II) with the amendment of CuO NPs. Meanwhile the nanoscale ferrihydrite has been proved to enhance the microbial dissimilatory iron reduction [49]. The transfer of an electron during dissimilatory iron reduction must occur via the electron shuttle or direct contact between iron minerals and microbial cells, and the latter pathway requires a distance less than 14 Å between minerals and cells [45]. Due to the small size effect, nanoparticles can significantly increase the contacting efficiency between iron oxide and microbial cells [50,51] and consequently enhance the dissimilatory iron reduction ability. Further combining the offsetting of the organic matter with the particle effects of MNPs and the different responses of Fe(II) in the two different soils, we considered that CuO NPs could increase the Fe(II) content by enhancing microbial dissimilatory iron reduction through their particle effects.

Although the content of organic matter is rather low, the functions of soil organic matter were closely related to the soil foundation, soil fertility, environmental protection, and sustainable development of agriculture. In this study, CuO NPs showed no significant effect on the TOC content of JSS, which was consistent with the results of Ben-Moshe et al., who claimed that small experiment systems could not meet all the conditions (e.g., exogenous oxidizing agents, lighting, etc.) for the permineralization of organic matter [52]. However, we found that CuO NPs could accelerate organic matter degradation to a certain degree, which was also supported by Ben-Moshe [53]. In an aqueous solution, CuO NPs were observed to increase the degradation rate of some organic pollutants as a catalyst, and the catalytic efficiency was proved to be positively related to the particles’ specific surface area instead of the metal ion dissolution of CuO NPs [53,54]. However, Zhou et al. suggested that metal ions dissolved from MNPs could combine with refractory organics to form soluble compounds [55]. Moreover, the strong oxidation activity of Cu^2+^ could trigger the Fenton reaction in the surface of microbes and produce active radicals to further affect organic matter oxidation [31,53]. Moreover, we found a stronger effect of CuO NPs on DOC in JSS than in HHS, which might have been caused by the different ion strengths in the two soils (data not shown). The metal ions in the soil could compete with the particles on the active sites of the reactions, thus mitigating the reaction of organic matter oxidation [56].

Soil microbes are the most complex and activated components in the soil, and most of the changes in the soil properties and components discussed above were induced and regulated by soil microbes. MBC is an indicator of soil microbial biomass. The results showed that the effect of CuO NPs on soil microorganisms was closely related to the exposed dosage, treatment time and soil environment. Interestingly, a low dosage of CuO NPs could result in an abnormal increase of MBC due to the theory of hormesis [57], leading to a dose-response phenomenon characterized by a low dose stimulation and high dose inhibition in toxicology. Hormesis of many traditional environmental pollutants has been widely reported, but results are still lacking for MNPs [11]. With the increase of CuO NPs, both the Cu ions and the nano-scale particles could cause damage to the microbial cells and result in the decrease of MBC [58]. Soil organic matter was considered to be protective of soil microbes, resulting in higher MBC and recovery of MBC in the long term in the HHS than for the JSS treated with CuO NPs. Similar results were observed by other studies [59,60,61]. The coating of soil organic matter on the surface of nanoparticles could reduce the direct contact between the microbial cells and the nanoparticles [59]. Moreover, the soil organic matter could reduce the microbial availability and toxicity of MNPs by complexing the metal ions [58,59]. Besides, with long-term exposure, the soil microorganisms would gradually adapt to the environmental stress, thus alleviating the toxicity of MNPs [62].

## 5. Conclusions

In this study, the complex interactions between the bioavailability and chemical forms of CuO NPs and paddy soil properties were observed. CuO NPs could transform rapidly after entering the paddy soil system and tended to form more stable precipitations and complexes. The amendment of CuO NPs increased soil pH, Eh, the contents of Fe(II) and dissolved organic matter while severely reducing the MBC in the soils during long-term flooding. Besides, the alternate drying–wetting process significantly affected the results, probably by changing the moisture and oxygen content. Either the CuO NPs transformations or their effects on the soil properties and components are dependent on the soil properties. The organic matter showed the potential to mitigate the negative effects of CuO NPs to some degree. In general, the input of CuO NPs significantly changed the physicochemical properties and components of paddy soil, which might be a potential risk to the paddy soil ecosystem. Thus, more attention should be paid to the effects of MNPs on the natural soil environment.

## Figures and Tables

**Table 1:** Fitting results of the Cu XANES spectra of soil samples exposed to 1000 mg/kg CuO NPs using a linear combination of the data for model compounds.

Reference Compounds	Percentages of Targeted Components (%)					
	JSS-0 Day	JSS-60 Days	JSS-90 Days	HHS-0 Days	HHS-60 Days	HHS-90 Days
CuO	69.3	0.0	0.0	88.4	0.0	41.9
Cu-goethite	15.2	0.0	0.0	24.3	0.0	0.0
CuS	19.9	0.0	0.0	2.5	0.0	0.0
Cu-humic acid	0.0	24.9	25.6	0.0	64.1	43.7
Cu(OH)_2_	0.0	11.1	0.0	0.0	39.9	19.0
Cu_2_S	0.0	38.7	40.3	0.0	2.6	0.0
Cu	0.0	27.5	35.4	0.0	0.0	0.0
R factor	0.0002	0.0001	0.0001	0.0005	0.0006	0.0003

## Supplementary Materials

The following are available online at http://www.mdpi.com/2079-4991/8/10/839/s1, Table S1: Basic physicochemical properties of the tested soils.
