# Supplementary material for: Effects of Copper Oxide Nanoparticles on Paddy Soil Properties and Components
Source: Nanomaterials (Basel). 2018 Oct 16;8(10):839. doi: 10.3390/nano8100839 (PMC6215298; doi:10.3390/nano8100839)
Supplement: Supplementary file 1 [file nanomaterials-08-00839-s001.docx]

**Supplementary data**

**Effects of copper oxide nanoparticles on paddy soil properties and components**

Materials and methods

**Soil culture experiment.** To maximally avoid the maldistribution of nanoparticles, CuO NPs and CuO BPs were amended through the method of step-by-step amplification: first, 1% (5g) of total soil was piled on a ceramic plate, exposed to the nanoparticles according to the ultimate concentration, and stirred until well combined. Then, the mixture of soil and nanoparticles was placed in a zip lock bag and shaken in a horizontal shaker for 1 h and sufficiently mixed with 10% (50g) of total soil. Finally, the mixture was mixed with the rest of the soil as per the above steps.

**Synchrotron radiation X-ray absorption fine structure (XAFS) analysis.** The Cu K-edge XAFS spectra of standard samples were recorded in beamline 14W1 in Shanghai Synchrotron Radiation Facility by the transmission mode, except for Cu adsorbed on goethite and Cu adsorbed on humic acid. These two references were recorded in the total fluorescence mode by a 19 element germanium solid-state detector. The spectra energy was calibrated using the K-edge of a copper foil, taking the first inflection point in the Cu K-edge as 8979 eV. The IFEFFIT Athena software was used to determine the pre-edge and post-edge line, calibrate the K-edge energy and normalize the spectra. Principal component analysis (PCA) and linear combination fitting (LCF) was proceeded to determine main forms of Cu.

**Table. S1.** Basic physicochemical properties of the tested soils

| Test items | Soil | |
| --- | --- | --- |
|  | (Jingshan Soil)JSS | (Heihe Soil)HHS |
| pH | 4.43±0.10 | 4.55±0.02 |
| Organic content (%) | 4.15±0.09 | 8.04±0.61 |
| Sand fraction (%) | 11.1±0.7 | 27.1±0.7 |
| Silt fraction (%) | 57.0±0.0 | 49.8±0.3 |
| Clay fraction (%) | 31.9±0.7 | 23.1±0.4 |
| Cation exchange capacity (cmol/kg) | 6.71±0.07 | 9.97±0.38 |
| Total Cu (mg/kg)**^a^** | 9.56±2.86 | 14.84±2.31 |
| Total S (mg/kg) | 349.9±58.7 | 420.4±4.0 |
| Total K(g/kg) | 21.26±4.16 | 19.76±0.46 |
| Total Na (g/kg) | 11.42±2.70 | 12.79±2.87 |
| Total Mg (g/kg) | 5.21±2.39 | 6.26±1.17 |
| Total Ca (g/kg) | 3.76±0.39 | 6.64±0.52 |
| Total Fe (g/kg) | 17.52±1.12 | 39.67±1.90 |
| Total Mn (mg/kg) | 193.24±18.14 | 596.80±8.40 |
| Total Zn (mg/kg)**^a^** | 100.83±5.59 | 107.19±8.60 |
| Total Pb (mg/kg)**^a^** | 124.79±9.19 | 122.68±10.25 |

**^a^**: Screen values of Cu, Zn, and Pb soil pollution risk on agricultural land in China are 50, 200, 80 (mg/kg), respectively.
